# Physicochemical Parameters and Bioaccessibility of Lactic Acid Bacteria Fermented Chayote Leaf (*Sechium edule*) and Pineapple (*Ananas comosus*) Smoothies

**DOI:** 10.3389/fnut.2021.649189

**Published:** 2021-04-07

**Authors:** Millicent G. Managa, Stephen A. Akinola, Fabienne Remize, Cyrielle Garcia, Dharini Sivakumar

**Affiliations:** ^1^Phytochemical Food Network Research Group, Department of Crop Sciences, Tshwane University of Technology, Pretoria, South Africa; ^2^QualiSud, Université de La Réunion, CIRAD, Université Montpellier, Montpellier SupAgro, Université d'Avignon, Sainte Clotilde, France

**Keywords:** gastrointestinal digestion, total phenols, carotenoids, antioxidant capacity, quality

## Abstract

In this study, popularly consumed traditional chayote leaves and locally produced pineapple fruit were used to develop a fermented smoothie using lactic acid bacteria (LAB) strains: *Lactobacillus plantarum* (*L75*), *Weissella cibaria* (*W64*), and their combination (*LW64* + *75*). The physicochemical parameters [pH, total soluble solids (TSS), and color], total phenols, and carotenoid contents of the smoothies fermented for 48 h and stored for 7 days at 4°C were compared with the unfermented (control) smoothies. Results indicated that LAB fermentation reduced the pH from 3.56 to 2.50 after 48 h (day 2) compared with the non-fermented smoothie at day 2 (pH 3.37). LAB strain *L75* significantly reduced the TSS content of the smoothies to 13.06°Bx after 2 days of fermentation. Smoothies fermented by *L75* showed overall acceptability after 7 days of storage compared with the non-fermented puree on day 0. The *LW64* + *75* significantly reduced the color change (Δ*E*), which was similar to the control. *L75* increased the phenolic content, and *W64* enhanced the total carotenoid content of the smoothies after 2 days of fermentation compared with other treatments. The use of an *in vitro* model simulating gastrointestinal (GI) digestion showed that fermentation with *L75* improved the total phenol recovery by 65.96% during the intestinal phase compared with the control. The dialysis phase mimicked an epithelial barrier, and 53.58% of the recovered free soluble are bioavailable from the *L75* fermented smoothies compared with the control. The antioxidant capacity of dialyzable fraction of the *L75* fermented smoothie was significantly higher than that of the control and smoothies fermented with *W64* or *LW64* + *75*.

## Introduction

Several authors have reported the beneficial roles of fruits and vegetables in preventing and managing chronic diseases, such as coronary heart diseases, stroke, obesity, diabetes, and cancer (1–3). The World Health Organization (4) recommends a minimum of 400 g of fruits and vegetables, or five portions per day, excluding starchy tubers, to ensure good health. The United States Department of Agriculture (USDA) (5) guidelines state that an individual must consume one cup (~237 g) of raw or cooked vegetables or two cups of raw leafy greens a day. However, fruits and vegetables are highly perishable and vulnerable to post-harvest losses, especially during the supply chain, thus affecting food security (6). Efforts are being made to reduce food loss at farm gate level and to profit the local economies by introducing agro processing products.

Fruit and vegetable products are valuable sources of fibers, antioxidants, and essential fatty acids (7). Fruit and vegetable juices are generally processed through juice extraction, followed by thermal processing for microbial stabilization. The thermal processing of juice can have negative effects on vitamins, such as ascorbic acid, thiamin, and folic acid (8). However, juice extraction is a processing step that removes insoluble dietary fibers, although these exert positive health effects (9). Therefore, another possible way for consumers to obtain the nutritional benefits from fruits and vegetables with less processing is through smoothies. Smoothies are fruit only or fruit and vegetable based semi-liquid nutrient-dense products with a smooth consistency (10).

Lactic acid fermentation is one of the most economical, oldest, and natural methods of food processing and preservation that keeps or enhances the efficiency and quality of foods while improving the organoleptic qualities and nutritional properties of the product (11). The importance of fermented products benefits the local economy and the communities in developing countries (12). Lactic acid bacteria (LAB) are a group of Gram-positive bacteria, which produce lactic acid as the main product of carbohydrate fermentation. During fermentation, the decrease in pH value and production of antimicrobial compounds by LAB enables the inhibition of spoilage and pathogenic microorganisms (11). LAB fermentation improves the content of riboflavin, folate, vitamin B12, sugar polymers, aroma compounds, or low-calorie polyols (mannitol, sorbitol) in substrates (13, 14). The lactic acid fermentation modifies phenolic composition and enhances the antioxidant activity in fermented tea extracts (15). Although lactic acid fermentation improves the antioxidant components in smoothies or fruit juices, it is important to know the potential availability of antioxidant components after digestion to evaluate its benefits (16).

The numerous nutritional and health benefits of chayote vegetable to consumers have encouraged growers of its cultivation and manufacturing of products by the local industry. Chayote leaves (*Sechium edule*) are one of the Réunioneses' favorite vegetables, which are locally naturalized (17). Chayote belongs to the Cucurbitaceae family and is familiar as mirliton, choko, chouchou (Jamaica), and chuchu (Brazil). The leaves are heart-shaped, 10–25 cm wide (18). A 100 g portion of young chayote leaves contains protein (4.0 g), fat (0.4 g), carbohydrates (4.7 g), fiber (1.2 g), Ca (58 mg), P (108 mg), Fe (2.5 mg), thiamin (615 μg), riboflavin (0.08 mg), niacin (0.18 mg), and ascorbic acid (1.1 mg) (19). Chayote leaf incorporation in product development holds huge potential due to its laden nutrient and biological functions, which could help improve healthy livelihoods, reduce wastage, and enhance agricultural sustainability. In contrast, pineapple (*Ananas comosus* cv. Queen Victoria) is an exotic fruit popularly grown in the Réunion Island and with recognized sensory value; a 100 g portion of the fruit contains protein (0.5 g), sugar (9.9 g), Ca (13.00 mg), K (109 mg), Fe (0.29 mg), Na (1 mg), fiber (1.4 g), total carbohydrate (13 g), and total fat (0.1 g) (5). Pineapples contain carotenoids, and their contents vary with cultivars and range from 29 to 565 μg/100 g on fresh weight (FW) basis (20). However, during the production, there is surplus supply of pineapples at the market, and cold storage facility is limited. Therefore, fermentation of pineapples and Chayote leaves was envisaged to resolve the post-harvest loss encountered during over supply.

Fermentation of fruit and vegetable substrates with desirable microorganisms could be a strategy to improve the nutritional quality, polyphenols, and antioxidant levels of fruit and vegetable products and could help to meet the nutritional and health needs of consumers. The use of LAB as starter cultures to enrich the biological value of foods has been reported (21). Another strategy to improve the nutrient quality of fruit smoothies could be by enrichment with indigenous vegetables or fruits. Polyphenols have been reported to confer health protective effects against cardiovascular and neurodegenerative diseases in humans (22). However, polyphenols from the diet must be bioaccessible for their bioactivity after undergoing an *in vivo* gastrointestinal (GI) digestion. The *in vitro* GI digestion is a reference tool to study the bioaccessibility of the dietary polyphenols (23). In the gastric digestion phase, a low pH environment, which is typical of the stomach's conditions, helps to stabilize and enhance the release of phenolics in phenolic–protein complex compounds (24). Tagliazucchi et al. (25) showed that only 62% of the original polyphenol content of grapes were bioaccessible after GI digestion. Bouayed et al. (23) suggested that the GI tract is an “effective extractor” for polyphenols present in food matrices and the polyphenols could be made available for absorption in the intestine. The use of *in vivo* human or animal models to investigate the GI tract requires an ethical clearance, is time consuming, and is expensive; therefore, *in vitro* digestion models are used to mimic the GI tract condition of humans during transit of complex food matrix and to investigate the bioaccessibility of compounds in food (26).

In light of the above, the objectives of this study were to investigate the efficacy of selected LAB strains, as standalone or in combination, to modulate the physicochemical properties of a fermented chayote leaf–pineapple smoothie, and to evaluate the total phenol and carotenoid contents, antioxidant capacity (ferric ion reducing antioxidant power, FRAP), and the bioaccessibility of phenolic compounds of the fermented smoothie after an *in vitro* gastric and intestinal digestion.

## Materials and Methods

### Chemicals

Culture media were purchased from Biokar Diagnostics (Solabia Group, Pantin, France) and Condalab (Madrid, Spain). Reagents were obtained from Sigma-Aldrich (Saint-Quentin-Fallavier, France) and VWR chemicals (Fontenay-sous-Bois, France). Type VI-B porcine pancreatic α-amylase, type I α-glucosidase from baker's yeast, starch, 4-nitrophenyl-β-d-glucuronide (pNPG), and voglibose for enzymatic experiments were also purchased from Sigma-Aldrich (Saint-Quentin Fallavier, France). The LAB cultures *Weissella cibaria* 64 (*W64*) and *Lactobacillus plantarum* 75 (*L75*) were obtained from the Microbiology Laboratory of QualiSud, Université de La Réunion, France (27).

### Plant Material and Smoothie Preparation

Pineapples (*A. comosus*) and chayote leaves (*S. edule*) were purchased from a local market in Réunion Island, France. Chayote leaves, free from dirt and damage caused by pests or decay, were selected, washed with tap water, and rinsed with distilled water. The leaves were dried on a paper towel, sliced into two halves, and homogenized with a domestic blender. Ripe pineapple fruits, free from signs of damage or decay, were peeled, cut using a knife, blended for 3 min, and then bottled in sterile glass containers. For 1.8 L of pineapple juice, 270 g of homogenized chayote leaves were added, and the mixture was blended to obtain a smoothie. The mixture was pasteurized at 82°C for 10 min using a water bath and then cooled to room temperature for 2 h.

### Starter Cultures and Fermentation of Smoothie

The LAB strains used in this study, *W. cibaria* (*W64*) and *L. plantarum* (*L75*), were obtained from the Microbiology Laboratory of QualiSud, Université de La Réunion, France (27). The strains were reactivated and propagated by successive suspension in 9 ml of de Man, Rogosa, and Sharpe (MRS) broth and incubated anaerobically for 48 h at 30°C in an incubator as previously reported by Nirina et al. (28). The resulting cells were harvested by centrifuging at 12,000 × g for 5 min at 4°C, cleaned, and suspended in sterile distilled water. The concentration of cultures was determined by the turbidity method on a UV–visible Spectrophotometer (SPECTROstar Nano; BMG LABTECH GmbH, Ortenberg, Germany). The concentration of pre-cultures was adjusted to 0.05 McFarland standard concentration (1–5 × 10^8^ CFU/ml) at 660 nm wavelength, and 1% inoculum was inoculated into the smoothies (29). Each strain alone and a combination were used to inoculate the smoothies. The combined starter (*W64* + *L75*) was developed at equal ratio (1:1; v/v). The smoothies were incubated at 37°C for 48 h and then stored for 7 days at 4°C. Thereafter, the fermented smoothies were stored at −20°C until analysis. The un-inoculated smoothie served as a control. The fermentation was performed in triplicate.

### Microbial Enumeration

The microbial load and LAB count of the samples were evaluated at the beginning, end of fermentation, and storage through the bacteria count using the pour plating techniques (30). Briefly, successive serial dilutions of smoothie sampled at 0, 2, and 7 days were made in sterile buffered peptone water and then plated on appropriate media. For the total bacterial and fungal (yeast and mold) and surviving LAB counts, dilutions were plated on Nutrient Agar (NA), Yeast Extract Glucose Chloramphenicol Agar (YGCA), and MRS plates, respectively. The MRS plates were incubated anaerobically at 30°C for 48 h, the NA plates at 37°C for 24 h, and the YGCA plates at 27°C for 5 days. The bacterial and fungal population were enumerated and expressed as colony forming units per ml (CFU/ml) of samples. Enumeration was done using five replicate plates for each sample.

### Physicochemical Properties

The physicochemical properties of the fermented and non-fermented smoothies were determined at 0 and 2 days of fermentation and 7 days of storage. The color of the fermented and non-fermented smoothies was determined using a CM-3500d spectrophotometer and analyzed using the SpectraMagic NX software (Konica Minolta, NJ, USA) to assess the effect of fermentation on the color. Measurements were made using L^*^, a^*^, and b^*^ color coordinates where L^*^ designates lightness, a^*^ is the color component from red to green, and b^*^ represents the component from yellow to blue (31). Total color difference (Δ*E*) was calculated according to Managa et al. (31), in which $L_{2}^{*}$, $a_{2}^{*}$, and $b_{2}^{*}$ refer to the assay condition, and $L_{1}^{*}$, $a_{1}^{*}$, and $b_{1}^{*}$ refer to the control smoothie.

The pH meter (pH2700 EUTECH Instruments, IL, USA) measured the pH of the fermented and non-fermented smoothies (10 ml). The measurement of the total soluble content of samples occurred before and after fermentation and storage, using the ATAGO PAL-3 pocket refractometer (ATAGO USA, Inc., WA, USA). The refractive index was recorded and converted to °Bx.

### Total Carotenoids

Samples obtained at 0, 2, and 7 days were evaluated for total carotenoid content. Briefly, 1.5 g of samples was homogenized with 5 ml of extracting solvent (hexane/acetone/ethanol, 50:25:25; v/v/v) and centrifuged at 3,000 × g for 5 min at 5°C. Then, 1 ml of hexane was added to the supernatant, and the absorbance was measured at 450 nm using a UV-180 Shimadzu spectrophotometer (Shimadzu, Buckinghamshire, UK). External calibration with a β-carotene standard solution was used, and total carotenoid content was expressed as mg/100 g dry weight (DW).

### Sensory Evaluation

The organoleptic properties of the smoothies were evaluated using the qualitative descriptive analysis method as previously described by Oliveira et al. (32) with slight modification. Seven trained panelists from a pool of healthy interested participants were used in the study, comprising both male and female participants. Participants were trained to identify the desired attributes in the smoothies prior to evaluating the test samples. The tasting was done in white light illuminated individual cubicles, and samples were presented chilled at standard room temperature. Two tasting sections were adopted in the study after the trained panelists have learnt to identify the desired attributes. The smoothies were scored using a structured scale ranging from 0 to 5 (0, absent; 1, weak; 5, strong). The reference samples used for each attribute are presented in Table 1. The cut-off point of 3 was set for acceptability for each attribute.

**Table 1:** Sensory evaluation of fermented and non-fermented smoothies.

**Smoothies**	**Color**	**Flavor**	**Consistency**	**Sourness**	**Sweetness**	**Overall acceptability**
	Ripe Pineapple juice + 1% food grade browning	Pineapple juice (100%)	Glucose syrup solution (10%)	Sweetened yogurt	Sucrose solution (70%)	Commercial fresh fermented smoothie
Non-fermented × D0	2.67 ± 33^ab^	2.67 ± 0.33^a^	3.33 ± 0.67^a^	2.33 ± 0.33^c^	4.67 ± 0.33^a^	1.67 ± 0.67^c^
Non-fermented × D2	2.67 ± 33^ab^	2.67 ± 0.33^a^	4.33 ± 0.33^a^	3.33 ± 0.67^abc^	4.33 ± 0.67^ab^	2.00 ± 0.58^bc^
W64 × D2	3.67 ± 33^a^	3.33 ± 0.67^a^	4.33 ± 0.67^a^	3.33 ± 0.67^abc^	3.67 ± 0.33^abc^	2.67 ± 0.33^abc^
L75 × D2	2.33 ± 33^bc^	3.67 ± 0.33^a^	4.33 ± 0.33^a^	4.33 ± 0.67^ab^	3.00 ± 0.58^bcd^	3.67 ± 0.33^a^
LW64 + 7 × D2	3.67 ± 33^a^	3.33 ± 0.67^a^	4.67 ± 0.33^a^	4.67 ± 0.33^a^	2.33 ± 0.33^cd^	2.33 ± 0.33^abc^
Non-fermented × D7	2.67 ± 33^ab^	2.67 ± 0.33^a^	3.67 ± 0.33^a^	2.67 ± 0.33^bc^	2.67 ± 0.33^cd^	1.67 ± 0.67^c^
W64 × D7	2.33 ± 33^bc^	2.67 ± 0.33^a^	4.00 ± 0.58^a^	2.33 ± 0.67^c^	2.33 ± 0.56^cd^	2.67 ± 0.33^abc^
L75 × D7	1.33 ± 33^c^	2.33 ± 0.33^a^	3.33 ± 0.33^a^	3.33 ± 0.33^abc^	2.00 ± 0.58^d^	3.33 ± 0.33^ab^
LW64 + 75 × D7	2.67 ± 33^ab^	2.67 ± 0.33^a^	4.33 ± 0.33^a^	3.67 ± 0.33^abc^	0.33 ± 0.58^e^	2.33 ± 0.33^abc^

*W64, Weissella cibaria 64; L75, Lactobacillus plantarum 75; LW64 + 75, W. cibaria 64 + L. plantarum 75; D0, day 0; D2, 2 days; D7, 7 days; non-fermented smoothies (control). Different superscript alphabets are significantly different along the columns (p ≤ 0.05)*.

### Total Phenolic Content

The Folin–Ciocalteu assay was used to measure the total polyphenol content of the fermented and non-fermented smoothies (11). Briefly, 100 μl of samples and 15 μl of Folin–Ciocalteu reagent were mixed in a 96-well plate and incubated for 4 min at 25°C; thereafter, 60 μl of 700 mM Na_2_CO_3_ was added to each well, and the mixture was incubated for 1 h in the dark. The absorbance was read at 760 nm using a microplate reader (Infinite M200 PRO; Tecan, Mannedorf, Switzerland). Gallic acid was used as a standard, and the results were expressed as gallic acid equivalents (GAE) in mg/g DW.

### FRAP Assay

Total antioxidant scavenging activity was determined using the method described by Llorach et al. (33). A 0.2 g sample of freeze-dried fruit puree was extracted using 2 ml of sodium acetate buffer (pH 3.6). In a microplate, 220 μl of FRAP reagent solution {10 mmol/l TPTZ [2,4,6-tris (2-pyridyl)-1,3,5-triazine]} was acidified with concentrated HCl and 20 mmol/l FeCl_3_, followed by 15 μl of the homogenized puree samples. The absorbance was read at 593 nm with a spectrophotometer (SPECTROstar Nano; BMG LABTECH GmbH, Ortenberg, Germany). The reducing antioxidant power was reported as Trolox, expressed in μmol Trolox Equivalent Antioxidant Capacity (TEAC)/100 g DW.

### *In vitro* Digestion of Smoothie

To evaluate the concentration of antioxidant compounds released and available for absorption from the smoothies, the simulated GI digestion method was adopted according to the Infogest nature protocols (34). All smoothie samples that were stored for 7 days at 4°C were subjected to successive gastric and pancreatic conditions. Briefly, 10 g of homogenized smoothie was mixed with simulated gastric fluid (SGF) to get a 40 ml of final volume, after the pH was adjusted to 3 with HCl and 10 ml of pepsin (2,000 U/ml) was added. The mixture was incubated at 37°C for 2 h under agitation. After the gastric phase, aliquots were collected for later analysis, and the reactions were stopped by cooling the test tubes on ice.

The intestinal phase was then divided into two successive steps: agitation step and dialysis process with tubing cellulose membrane (MWCO 10 kDa) as a simplified model of the epithelial barrier. Gastric phase sample was mixed with simulated intestinal fluid (SIF) to get an 80 ml of final volume after addition of pancreatin (100 U/ml) and bile salt (10 mM) and pH adjustment at 7. This mixture was then incubated for 2 h at 37°C. After the intestinal phase, aliquots were collected for later analysis. Dialysis bags filled with 5.5 ml NaCl (0.9%) and 5.5 ml NaHCO_3_ (0.5 M) sealed with clips were completely immersed into the GI digested immediately after digestion. The samples with dialysis bags were then incubated for 45 min at 37°C under agitation. Aliquots were collected for later analysis at the end of the incubation time.

### Statistical Analysis

The conducting of the experiment was in a completely randomized design, with three replicates per treatment and repeated twice. The software XLSTAT (Addinsoft, Paris, France) was used for all statistical analyses. Data were subjected to one-way analysis of variance (ANOVA), and significant effects of factors were detected with a Fisher's test (*p* < 0.0001). The mean scores from sensory analysis were calculated, and the significant differences were evaluated by ANOVA, followed by *post-hoc* using Duncan Multiple Range test (*p* ≤ 0.05).

## Results and Discussion

### pH, TSS, and Changes in Color

**Figure 1:**
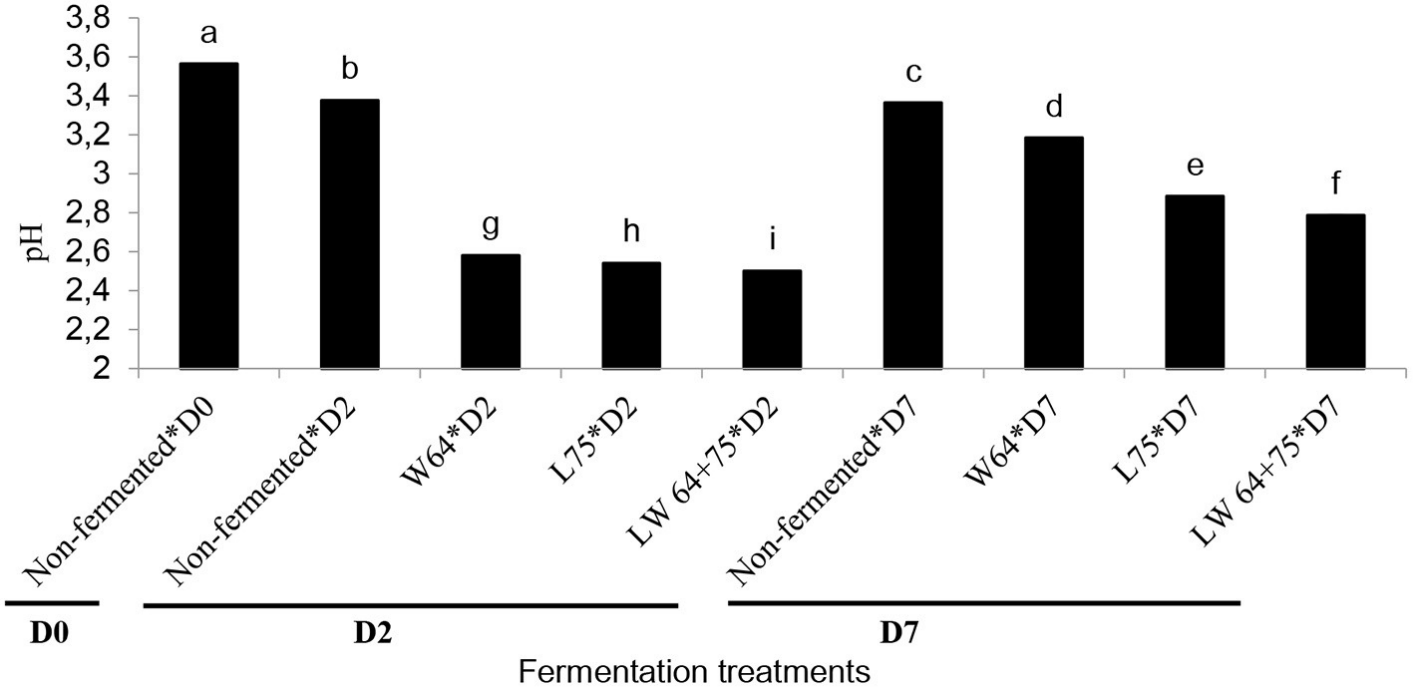
Changes in pH value during lactic acid fermentation of smoothies. Bars with different alphabetic letters are significantly different at *p* < 0.0001 level. *W64, Weissella cibaria* 64; *L75, Lactobacillus plantarum 75*; *LW64* + *75, W. cibaria 64* + *L. plantarum* 75; D0, day 0; D2, 2 days; D7, 7 days; non-fermented smoothies (control).

Food acidification is the primary mechanism involved in lactic acid fermentation to preserve and ensure the safety of foods by preventing the growth of spoilage and pathogenic microorganisms in fermented food. Lactic acid is a major metabolite in the homo-lactic fermentation and could serve as a preservative in fermented foods (35, 36). In this study, the initial pH value of the smoothie was 3.56; after 2 days of fermentation, the pH value ranged from 2.50 to 2.54, which was lower than the non-fermented smoothie (3.37). The lowest pH (2.5) was obtained in the smoothie fermented with the combination of *LW64* + *75* at 2 days (Figure 1). The pH values of the fermented smoothie dropped after 2 and 7 days of fermentation, thus supporting the previous report of a decrease in pH of *L. plantarum* fermented tomato juice (37). In addition, the least pH decrease (pH value of 3.18) was in the smoothie fermented with *W64*. Similar observations with a lesser decrease of pH in *W64* were reported in pineapple juice (11). However, after storage for 7 days at 4°C, the pH values of the fermented smoothies significantly increased compared with the pH values at 2 days. The smoothie fermented with combined starters (*LW64* + *75*) had the lowest pH value (2.50); this is probably due to the induced fermentation with LAB starters that could have reduced the pH during the first 2 days of fermentation, and thereafter the increased pH during storage might be due to the onset of metabolic activities of colonizing bacteria or fungi that utilize lactic acid as a carbon source, thus releasing metabolites that could alter the acidity of foods (30). The higher pH in the stored samples suggests that the LAB cultures could not survive for 7 days at the stored temperatures. Malic and citric acids are the main organic acids in pineapple (38) and could have been responsible for the acidity in the non-fermented smoothie. During the fermentation process, LAB metabolized simple sugars, such as sucrose, fructose, and glucose into organic acids, mainly lactic acid, and carbon dioxide leading to pH decrease (11).

**Figure 2:**
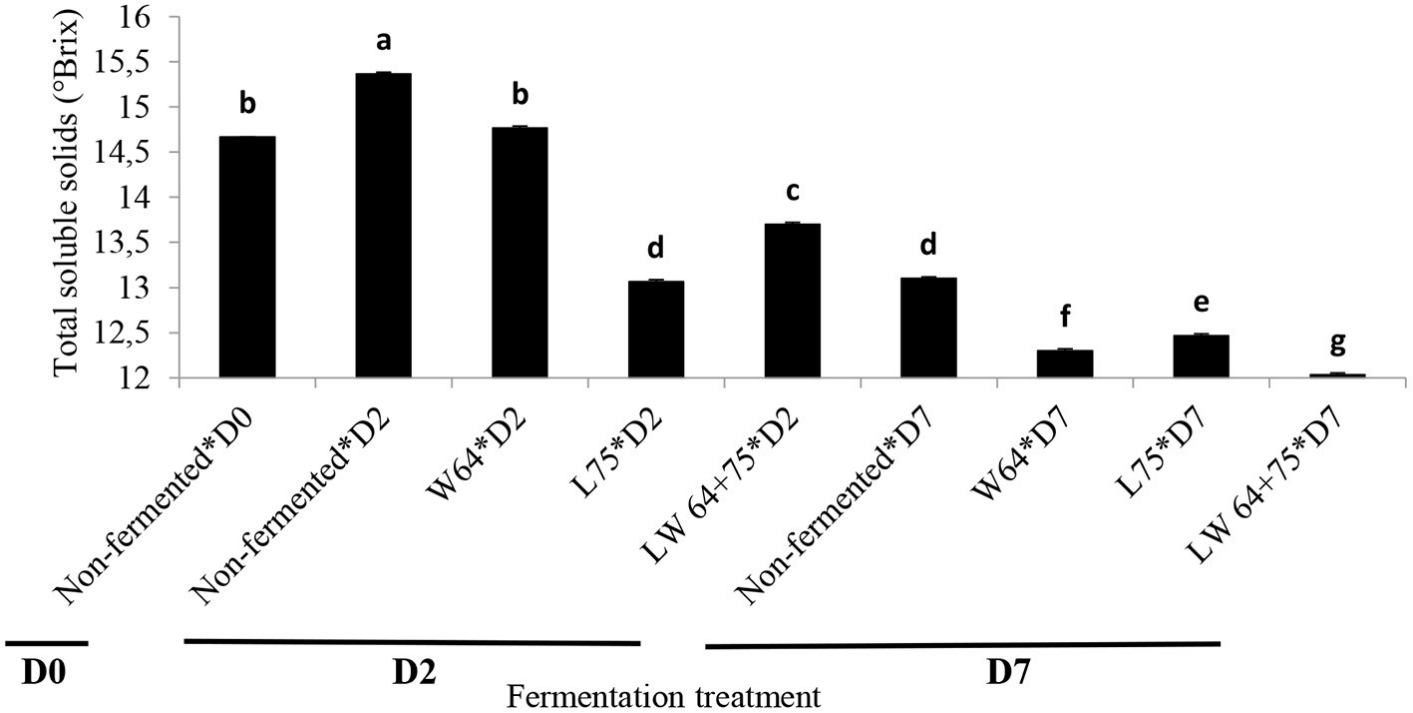
Changes in total soluble solids during fermentation of smoothies. Bars with different alphabetic letters are significantly different at *p* < 0.0001 level. *W64, Weissella cibaria* 64; *L75, Lactobacillus plantarum 75*; *LW64* + *75, W. cibaria 64* + *L. plantarum* 75; D0, day 0; D2, 2 days; D7, 7 days; non-fermented smoothies (control).

Total soluble solids (TSS) are important quality indicators that relate to sweetness (39), often referred to as sugar index. An initial value of 14.7°Bx was obtained in the smoothies, ranging from 13.1 to 14.8°Bx in the fermented smoothies after 2 days of fermentation (Figure 2). As expected, the TSS content of the smoothies significantly (*p* < 0.0001) decreased during lactic acid fermentation compared with the control (Figure 2). However, the smoothies fermented with *W64* showed the highest TSS (14.8°Bx) after 2 days of fermentation and were not significantly different to the control at the start of fermentation, whereas the *L75* and the combination (*LW64* + *75*) significantly (*p* < 0.05) reduced the TSS of the smoothie to 13.1 and 13.7°Bx, respectively, after 2 days. A decline in TSS content during fermentation is due to the utilization of sugars in smoothies by LAB strains for metabolism, cellular growth, and bioconversion into lactic acid (40). After 7 days of storage, the TSS content of the control smoothie decreased to 13.1°Bx, and a further decrease in TSS was observed in the smoothies fermented with *W64, L75*, and *LW64* + *75*. The smoothies fermented with the combined starter cultures (*LW64* + *75*) showed the lowest TSS content after storage. This suggests a continuous fermentation in the fermented smoothies and the non-inoculated sample resulting in the use of sugars by fermenting and colonizing cultures, respectively, at 4°C; hence, lactic acid fermentation of pineapple–chayote smoothies can still take place at cold temperature. The TSS decrease in the smoothie fermented with *LW64* + *75* represents 18% of the initial TSS value. The decrease in TSS after fermentation and storage supports the previous assertion in this study that lactic acid fermentations proceed in the fermented smoothies after cold storage. The decrease in TSS suggests the utilization of soluble sugars by LAB in the fermented smoothies, thus corroborating the report on the decrease in TSS of *L. plantarum* fermented cashew–apple juice after 72 h of fermentation (41) and in sugarcane and beet juice (42). Most LAB follow the Embden–Meyerhof, tagatose-6-phosphate, Leloir, or phosphoketolase pathways to synthesize lactic acids and carbon dioxide from soluble sugars in substrates, thereby causing a decrease in the pH by the metabolite (43). From a nutritional point of view, the decrease in sugar content of the fermented smoothies is an advantage over the non-fermented one in managing diabetic conditions, especially when all reducing sugar contents are transformed during fermentation (44).

**Figure 3:**
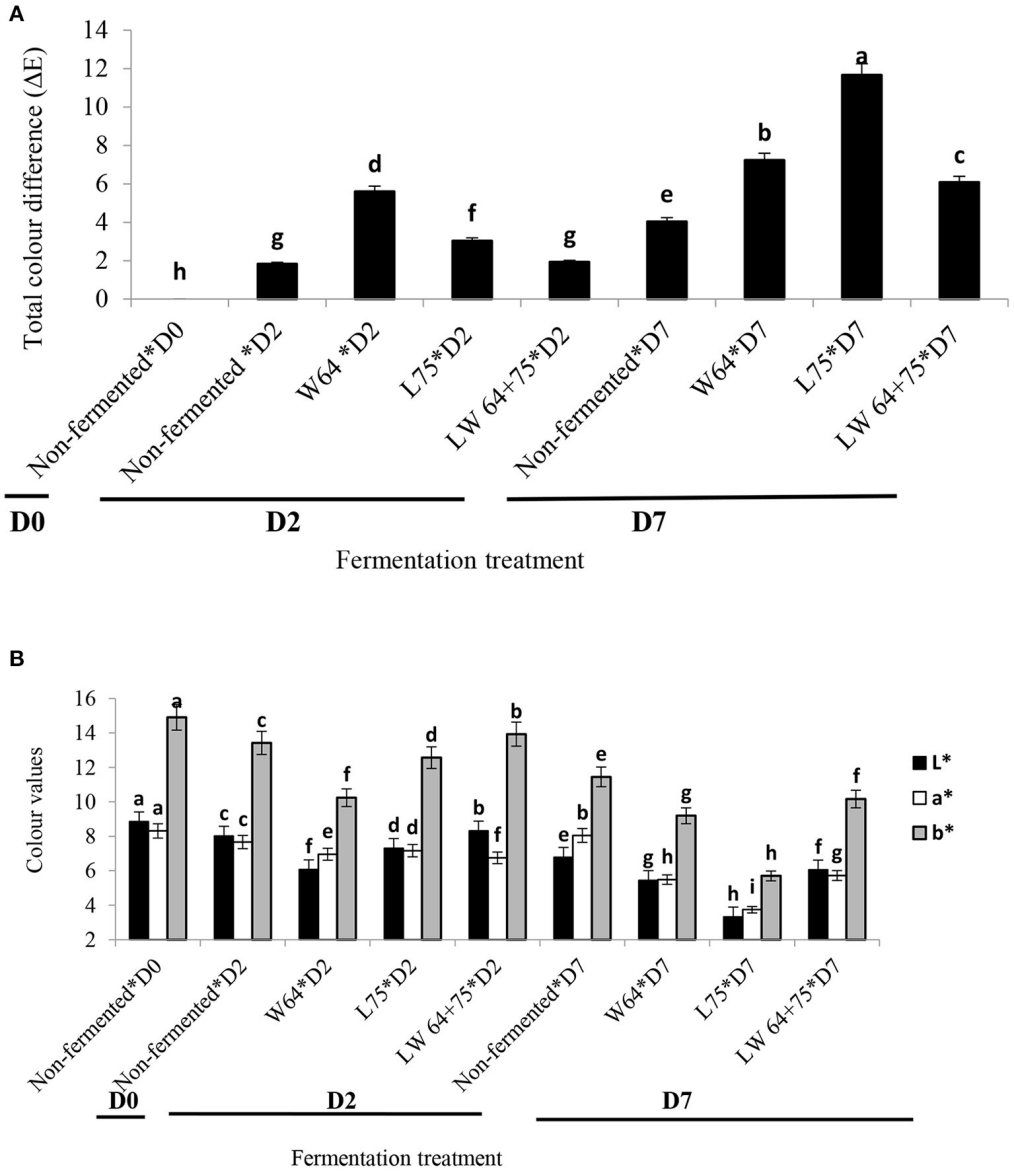
**(A)** Total color difference (Δ*E*) of lactic acid fermented smoothies. Bars with different alphabetic letters are significantly different at *p* < 0.0001 level. *W64, Weissella cibaria* 64; *L75, Lactobacillus plantarum 75*; *LW64* + *75, W. cibaria 64* + *L. plantarum* 75; D0, day 0; D2, 2 days; D7, 7 days; non-fermented smoothies (control); Δ*E* = change in color. **(B)** Color attributes (l*, a*, b*) of lactic acid fermented smoothies. Bars with different alphabetic letters are significantly different at *p* < 0.0001 level. *W64, Weissella cibaria* 64; *L75, Lactobacillus plantarum 75*; LW*64* + *75, W. cibaria 64* + *L. plantarum* 75; D0, day 0; D2, 2 days; D7, 7 days; l*, lightness; a*, redness/greenish; b*, yellowish/bluish; non-fermented smoothies (control).

Color is one of the most important food parameters since it influences consumer acceptability (45). As shown in Figure 3, color values of the smoothie varied according to fermenting LAB strains. Non-fermented (control) smoothie color parameters showed initial values corresponding to dark brown, and the parameter values decreased with time. The combined starter culture (*LW64* + *75*) fermented smoothies showed a significantly higher L^*^ (luminosity) and b^*^ values, whereas *L75* showed the highest a^*^ value after 2 days of fermentation. The higher redness to greenness color attributes in the *W65* fermented smoothie could be due to the steering activity of fermenting LAB causing enzymatic oxidation during the fermentation process. However, the period of fermentation significantly (*p* < 0.0001) influenced the color parameter values for all smoothies, especially in samples fermented with *W64* (Figure 3A). After 7 days of storage, the lowest values were observed in the *L75* fermented smoothie. The Δ*E* relates to the color difference of the smoothies fermented with *W64, L75*, or *LW65* + *75*. The Δ*E* of the fermented smoothies on day 2 ranged from 1.92 to 5.60, was the lowest in the smoothies fermented with *LW65* + *75* (1.92), and was significantly comparable to the non-fermented smoothies. The *W64* fermented smoothie (5.60) had the highest Δ*E*. According to Wang et al. (46), a color difference equal to 2 or greater (Δ*E* ≥ 2) is regarded as a significant color change in samples. Similarly, the color difference significantly (*p* < 0.0001) increased with prolonged storage compared with the control samples at day 7 (Figure 3B). The continuous color change could be attributed to an extended biochemical degradation and acidity caused by the fermenting cultures in the smoothies at a lower temperature. The lower color change in the combined starter culture treatment may be due to a dominating activity of the homo-fermentative LAB (*L. plantarum*) that blocks the enzymatic degradation in the fermented smoothies. *L. plantarum* has been identified as a strong homolactic fermenter of food substrates (47). Dark colors in foods negatively affect the consumer acceptability of food products (48). Despite the color modifications during fermentation, the color change in the smoothies fermented with combined starter is still comparable to the non-fermented at 2 days of fermentation.

### Impact of Fermentation on Sensory Attributes

The produced smoothies were evaluated for their organoleptic characteristics before and after fermentation and storage for 7 days. Table 1 presents the qualitative descriptive sensory evaluation of the fermented and unfermented smoothies. The color acceptability of smoothies ranged from slightly dark color (1.33) in *L75* smoothies stored for 7 days to moderately bright yellow color associated with pineapple juice in *W64* and *L75* smoothies (3.67) at 2 days of fermentation. The fermented smoothies (*W64* and *LW64* + *75*) at 2 days of fermentation were not significantly different at *p* ≤ 0.05 and also in the non-fermented samples at 0, 2, and after 7 days of storage. The lower values obtained for the fermented smoothies could be due to impaired lightness in the smoothies due to the addition of chayote leaves. The color score of samples were adjudged to be lower after storage of the smoothies. There was no significant difference in the flavor and consistency attributes of both fermented and non-fermented smoothies at 0, 2, and after storage for 7 days. However, the consistency in smoothies was adjudged as highly acceptable, whereas the flavor of the smoothies fermented for 2 days had an acceptable flavor compared with other samples. The perception of acid taste in the samples was scored the highest in *LW64* + *75* (4.67) followed by *L75* (4.33) at day 2 of fermentation and was significantly different to the non-fermented smoothies at day 0. The high perception of acid taste might be related to the high acid production by *L75*. *L. plantarum* is known to produce organic acids, with lactic acid being the major one during its biochemical degradation of sugar substrates (11); hence, the high acid taste in *LW64* + *75* could be due to a synergistic effect of acid production by the LAB strains, thus supporting the low pH obtained in this sample. The sweetness ranged from no sweetness (0.33) in *LW64* + *75* to high sweetness (4.67) in non-fermented samples at day 0. The sweetness perception reduced with fermentation and storage. The sample *L75* fermented smoothie after 2 days had the highest overall acceptability (3.67) compared with other smoothies and was significantly different to the non-fermented smoothies (*p* ≤ 0.05) at 0, 2, and after 7 days of storage.

### Survival of LAB in Smoothies

**Figure 4:**
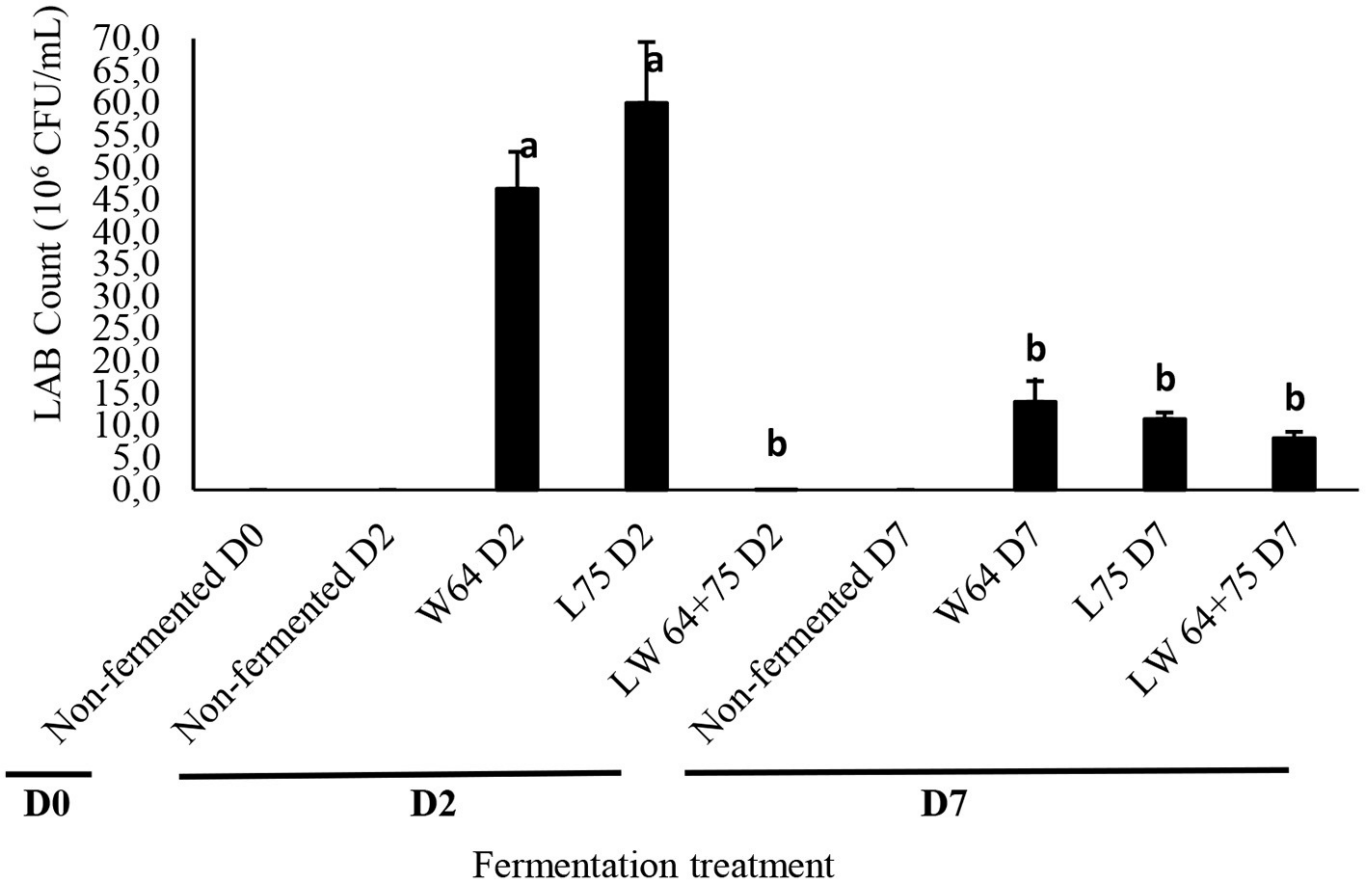
Surviving LAB count in fermented smoothies. Bars with similar alphabetic letters are not significantly different at *p* < 0.0001 level. *W64, Weissella cibaria* 64; *L75, Lactobacillus plantarum 75*; *LW64* + *75, W. cibaria 64* + *L. plantarum* 75; D0, day 0; D2, 2 days; D7, 7 days; non-fermented smoothies (control).

To assess changes in microbial quality, yeasts and mold growth were used as hygienic indicators to evaluate if the preparation was not contaminated. The microbial quality of the fermented smoothies was observed from the start of fermentation (day 0) and after 7 days (2 days of fermentation at 37°C plus 5 days of storage at 4°C) for the LAB strains, its combination, and control. From the data obtained, the yeast, mold, and aerobic bacterial population (not shown) remained below the detection level for all samples, and there were no significant differences in the treatments, thus meeting the safety of the product for consumption. The LAB counts in the fermented and non-fermented smoothies ranged from 0.01 to 60 × 10^6^ CFU/ml and were the highest in *L75* (60 × 10^6^ CFU/ml) but not significantly different to *W64* at day 2 of fermentation (Figure 4). However, the smoothies fermented with *LW64* + *75* had lower surviving LAB at day 2 and were significantly different to *W64* and *L75*. This might be due to a competition among the inoculated starters for dominance of the fermentation, thereby limiting the proliferation of LAB cells and death of the weaker ones. There was no detectable LAB growth in the non-fermented samples. Furthermore, after storage for 7 days, the LAB counts in the fermented smoothies ranged from 1.0 to 3.21 × 10^6^ CFU/ml without a significant difference between *W64, L75*, and *LW64* + *75* compared with the non-fermented smoothies at days 0 and 7. Low pH reportedly affects the survival of bacteria significantly in stored fruit juices (49). Hence, the higher survival of the LAB at day 2 with much lower acidity signifies the acid tolerance of *L75* and *W65* cultures, whereas the reduced LAB count at storage for 7 days signifies the non-survival of LAB at higher pH ≥3. Šeme et al. (50) had earlier reported an increase in the survival of *L. plantarum* KR6 cells in acidified medium (pH 2) and *L. plantarum* NCIMB 8826 in cranberry juice (51). The change in survival of LAB after storage for 7 days might be associated with the changes in the membrane fatty acids composition of the LAB cells, since it is important in regulating the proton permeability of the cell membranes (52).

### Changes in Phenolic Content and Total Carotenoid After LAB Fermentation

**Figure 5:**
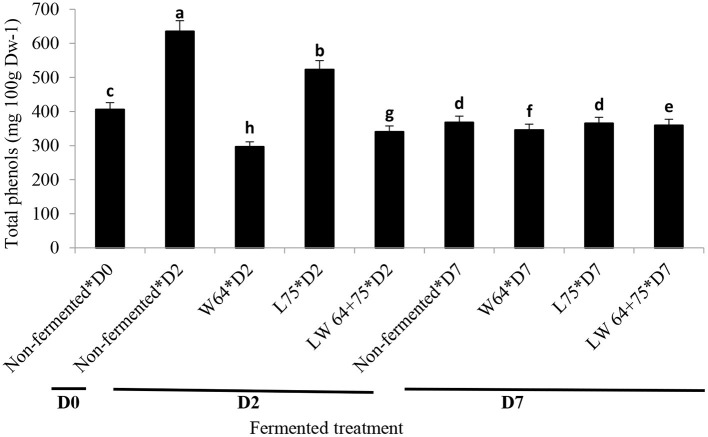
Changes in total polyphenol content (TPC) during fermentation of smoothies. Bars with different alphabetic letters are significantly different at *p* < 0.0001 level. *W64, Weissella cibaria* 64; *L75, Lactobacillus plantarum 75*; *LW64* + *75, W. cibaria 64* + *L. plantarum* 75; D0, day 0; D2, 2 days; D7, 7 days; DW, dry weight; non-fermented smoothies (control).

Total phenolic content (TPC; 634.7 mg/L) in the non-fermented smoothie was the highest on day 2 compared with the fermented smoothies and the control samples at day 2 or 7 (Figure 5). However, the smoothies fermented with *L75* for 2 days showed higher concentration of TPC than the smoothies fermented with *W64* or *LW64* + *75*. LAB reportedly hold the ability to convert food matrixes into functional moieties (41). The bioconversion of aglycone from phenolic glycosides by LAB can be strain-dependent (53), based on the specific enzymes. *L. plantarum* produces different enzymes, such as β-glucosidase, lactate dehydrogenase, amylase, peptidase, decarboxylase, phenol reductase, phenolic acid decarboxylase, and proteinase (54), which aided the increase or stabilization of phenolic content in smoothies after fermentation.

Ferulic acid, caffeic acid, quercetin, myricetin, kaempferol, and chlorogenic acid have been identified in chayote leaves, with chlorogenic acid (5.80 ± 0.12 mg/100 g FW) being the most abundant phenolics (55). Similarly, according to Wen and Wrolstad (56), raw pineapple juice contains N-l-γ-glutamyl-S-sinapyl-l-cysteine, S-sinapylglutathione, S-sinapyl-l-cysteine, furanones, glycosides, and p-coumaric acids. The TPC reduced significantly (*p* < 0.0001) in the fermented and non-fermented smoothies after storage (day 7). At day 7 after storage, the smoothies fermented with *L75, W64*, and *LW64* + *75* showed 12–16% reduction in the TPCs compared with the control (day 0). The non-fermented smoothie at day 7 after storage exhibited a 9% decrease in TPC compared with day 0. The decrease in TPC of the fermented smoothie is in agreement with the report by Hashemi et al. (57) in *L. plantarum* fermented sweet lemon juice after storage. Most processing steps, such as freezing, freeze-drying, and pasteurization, cause a large degradation of phenolic compounds in fruit (58, 59). Contrarily, lactic acid fermentation is recognized as a way to preserve TPC (60, 61). The observation made in this study corroborates the previous report on the increase in TPC after fermentation. Hydrolysis of complex glycosides during fermentation might have contributed to the increased phenolic contents. Hydrolysis could cause a cleaving of the phenolic–sugar glycosidic bonds or decarboxylation process that could help in the release of aglycones, degradation of gallotannins to simple phenolic acids, or formation of new phenolic compounds, such as pyrogallol, which could have reacted better with the Folin–Ciocalteu reagent leading to higher values of total phenolics (53, 62).

**Figure 6:**
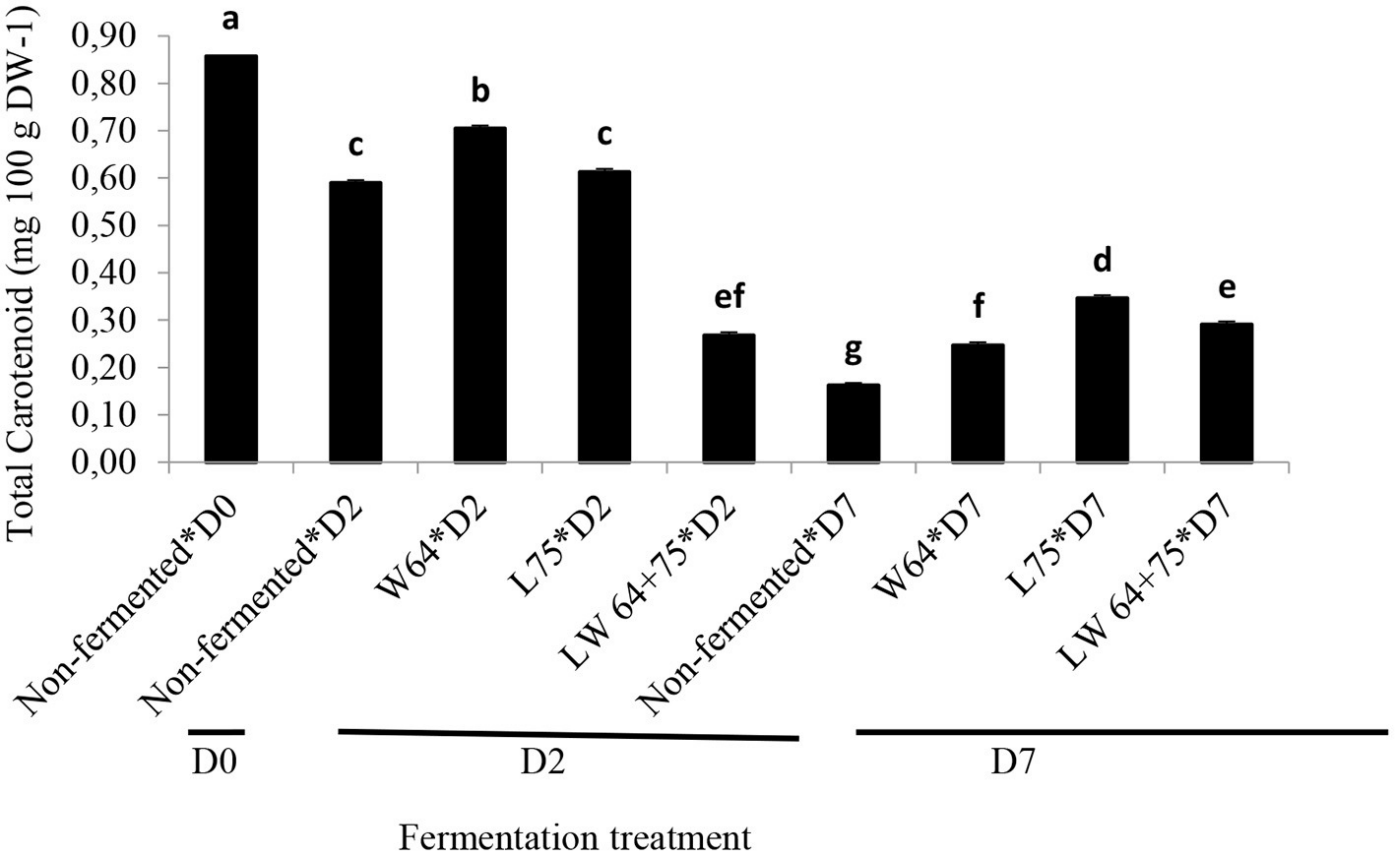
Changes in total carotenoid content during fermentation of smoothies. Bars with different alphabetic letters are significantly different at *p* < 0.0001 level. *W64, Weissella cibaria* 64; *L75, Lactobacillus plantarum 75*; *LW64* + *75, W. cibaria 64* + *L. plantarum* 75; D0, day 0; D2, 2 days; D7, 7 days; DW, dry weight; non-fermented smoothies (control).

The non-fermented smoothie (day 0) showed the highest total carotenoid contents. The effect of lactic acid fermentation on the carotenoids is highly dependent on the matrix, carotenoid composition, and LAB strain, but in general, the total carotenoid content remains stable (63). A gradual decrease of the carotenoid content of the non-fermented smoothie was observed with time of fermentation and storage. After storage, only 19% of the initial carotenoid content was present; however, on day 2 of fermentation, the decrease in total carotenoid contents of the *W64* fermented smoothie was lower than those of the *L75, LW64* + *75*, and control (Figure 6). The reduction in total carotenoid content of the smoothies after fermentation and storage has been reported in *L. plantarum* fermented sweet lemon juice (41). After 7 days of storage, the total carotenoid content of the fermented smoothie was significantly higher than that of the control at the same stage, and 29–40% of the initial carotenoid content remained. The carotenoid level in Queen Victoria pineapple is the highest among several cultivars, having 565 μg/100 g of pulp (64) and mostly composed of β-carotene. In leafy vegetables, carotenoids are mostly located in chloroplasts (64). The content of carotenoids generally decreases with processing of foods, due to isomerization, oxidation, and light degradation. However, the bioavailability of carotenoids, which is usually low in leafy vegetables, can be increased by processing steps, such as homogenization and thermal treatment (64, 65), since unbounding from complex structures and food composition, especially in lipids and fibers, plays a key role in carotenoid bioavailability.

### *In vitro*-Simulated GI Digestion Analysis

**Figure 7:**
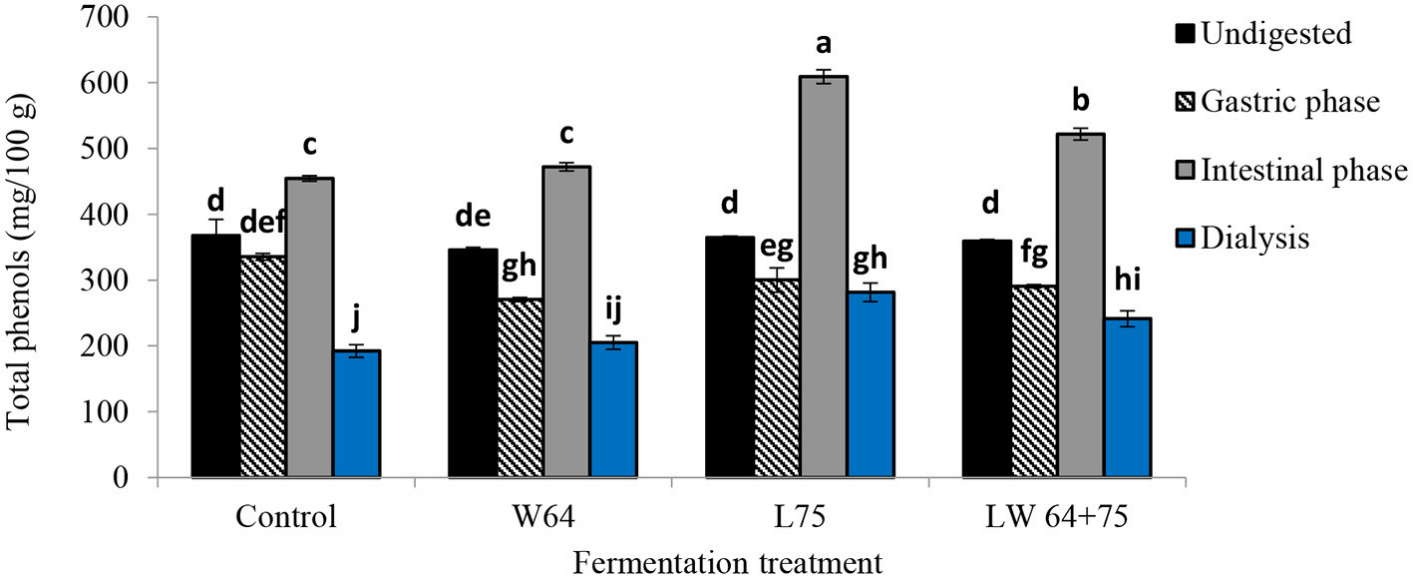
Total polyphenol content of LAB fermented smoothies subjected to simulated *in vitro* gastrointestinal digestion. Bars with similar alphabetic letters for a specific digestive phase are not significantly different at *p* < 0.001 level. *W64, Weissella cibaria* 64; *L75, Lactobacillus plantarum* 75; *LW64* + *75, W. cibaria 64* + *L. plantarum* 75. DW, dry weight; control, non-fermented smoothies.

Figure 7 illustrates the effect of GI digestion on total phenolic compounds from the fermented and non-fermented smoothies after 2 days compared with the undigested smoothies. The TPC in the non-fermented smoothies and fermented smoothies before digestion varied between 345.70 and 368.06 mg/100 g. The TPC in the gastric digesta of all fermented smoothies increased significantly (*p* < 0.0001) compared with their respective undigested sample; however, the non-fermented smoothies showed a non-significant difference (*p* > 0.0001). Moreover, the TPC in the intestinal phase was higher than that in the respective undigested samples and gastric digesta for all fermented and non-fermented smoothies, whereas the smoothies fermented by *L75* showed the highest concentration at 609.17 mg/100 g, followed by *LW*64 + *75* at 521.86 mg/100 g. A significant declining trend was observed in TPC at the dialysis phase compared with the undigested samples, gastric, and intestinal digesta. The bioaccessible TPCs after GI digestion at the dialysis phase were 77.15, 67.14, 59.32, and 52.23% in the smoothies fermented by *L75, LW64* + *75, W64*, and non-fermented smoothies, respectively, in terms of the percentage recovery relative to the TPC in the respective undigested samples.

A shift from the acid gastric condition to mild alkaline at the intestinal phase was expected to reduce the levels of bioaccessible total phenols as previously described in fruit juices (23, 66). However, an opposite trend was noticed with a higher but less pronounced level of total phenols in digested apple varieties, such as Jonaprince, Jonagold, and Golden, in the intestinal phase than in the gastric phase (23). During *in vitro* digestion of edible leaves, *Olax zeylanica* (“mella”), *Centella asiatica* (“gotukola”), and *Sesbania grandiflora* (“kathurumurunga”) showed higher levels of total polyphenol content in the intestinal phase than in the gastric digesta (67). The observed increase of TPC in the intestinal smoothie digesta compared with the gastric phase with respect to the undigested sample may be due to an increased release of phenolics bound to the matrix due to the activity of the intestinal digestive enzyme (pancreatin) (23), or to the phenolic interaction with cell wall carbohydrates, such as pectin present in smoothie, obstructing the solubilization of the phenolic compounds during gastric digestion (68). Furthermore, the decrease in pH during fermentation could have increased the extractability of phenolic compounds and their stability (23), as they are more stable in more acidic conditions. Additionally, it was shown that some phenolic compounds showed specific higher bioaccessibility at the intestinal phase than at the gastric phase, such as in p-coumaric acid and quercetin from *Moringa oleifera* leaves (69). The amount of released total phenolic compound (in terms of % recovery) was high in the intestinal digesta of the smoothies fermented with *L75* compared with those fermented with *W64* (*LW75* + *64*), control (non-fermented smoothies), and the undigested smoothies, indicating the effect of LAB strain on smoothie phenolic contents.

The degree of hydrolysis of glucosides affects the bioavailability of phenolic components related to their bioactivities (53). Li et al. (70) reported a similar observation during *in vitro* digestion of grape marc freeze-dried powder in the presence of probiotics. The increase in total phenols in the gastric and intestinal phases can be attributed to the conversion of simple phenolic compounds due to the glycosylation or decarboxylation or the depolymerization of high-molecular-weight phenolic compounds (70, 71).

The dialyzable fraction mimics the fraction that will be available for absorption into the systematic circulation by passive diffusion, which is a provisional approach to project the degree of intestinal epithelium absorption (68). The amount of dialyzable phenolics was lowest (192.25), and 52.23% recovery was related to the original undigested sample in the unfermented smoothies. Bouayed et al. (23) reported a similar trend in different apple cultivars. Almost 77.15 and 67.14% of free soluble dialyzable polyphenols were available for passive absorption in the *L75* and *LW75* + *64* fermented smoothies, respectively. Our results suggest that although the majority of the polyphenol compounds are available in the intestinal phase, the amount accessible at the dialysis phase was low. However, fermenting the smoothies with *L75* improved the bioaccessibility of total phenols compared with the smoothies fermented by *LW64* + *75* or *W64* or non-fermented smoothies.

**Figure 8:**
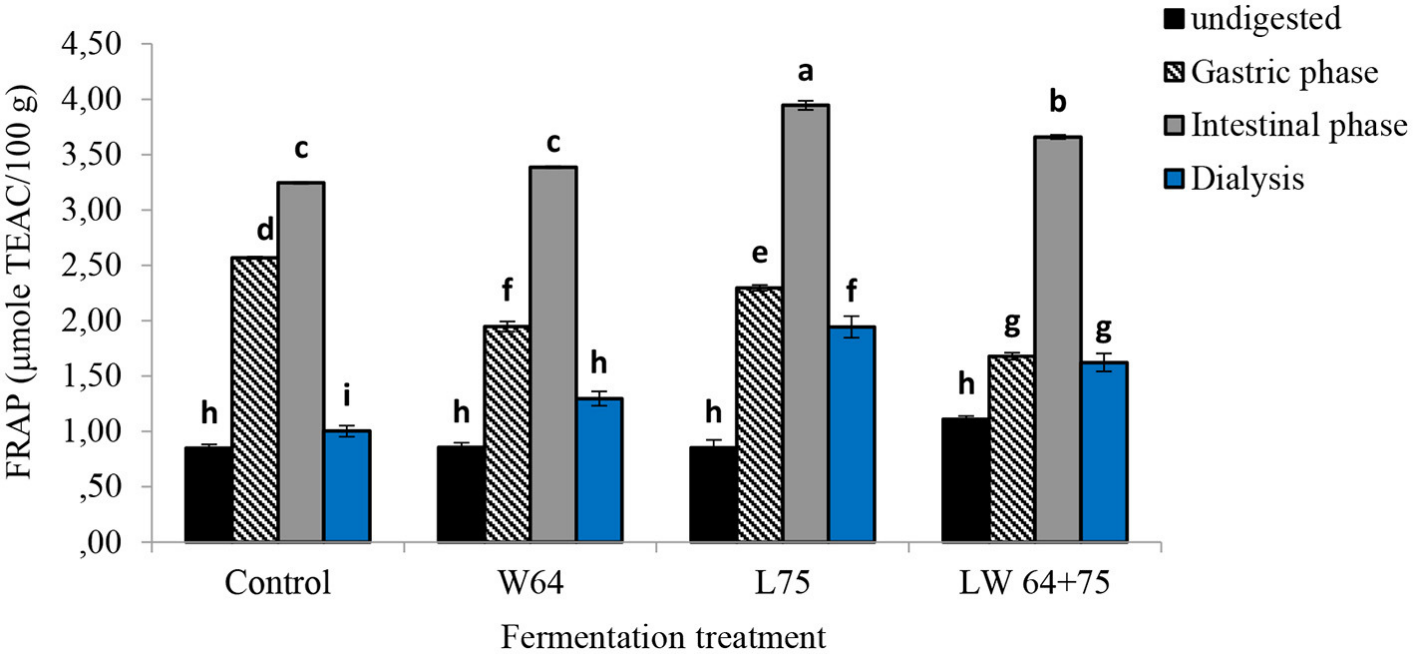
Ferric ion reducing antioxidant power (FRAP) of LAB fermented smoothies subjected to gastrointestinal digestion and dialysis. Bars with similar alphabetic letters for a specific digestive phase are not significantly different at *p* < 0.001 level. *W64, Weissella cibaria* 64; *L75, Lactobacillus plantarum* 75; *LW64* + *75, W. cibaria 64* + *L. plantarum* 75; DW, dry weight; control, non-fermented smoothies.

Antioxidant capacity (FRAP activity) in smoothies at the gastric phase was significantly higher than that in the fermented and non-fermented smoothies with respect to their original unfermented samples (Figure 8). Although the antioxidant capacity of the intestinal digesta of all non-fermented and fermented smoothies increased significantly compared with their respective undigested smoothie, the digesta of the smoothies fermented by *L75* showed the highest antioxidant capacity (3.94 μmol TEAC/100 g), followed by *LW64* + *75* (3.66 μmol TEAC/100 g). At the same time, the dialyzable digesta of the smoothies fermented by *L75* showed the highest antioxidant capacity (1.94 μmol TEAC/100 g) compared with undigested samples and the dialyzable digesta of the smoothies fermented by *W64* and *LW64* + *75*.

The observation confirms the increase in free soluble antioxidants responsible for antioxidant capacity compared with their corresponding non-fermented smoothie. There was a similar trend noted in the increase in total phenol and antioxidant capacity of the smoothies fermented with *L75* in the gastric and intestinal digesta, indicating positive correlations between total phenolics and total antioxidant capacity (24). At the same time, the transition from acidic (gastric) to alkaline (intestine) pH also could have played a role in boosting the antioxidant capacity of phenolics that could facilitate the deprotonation of the hydroxyl moieties in aromatic rings (23). Bouayed et al. (23) further suggested that the FRAP assay (performed at a pH of 3.6) could be more relevant to evaluate the antioxidant capacity in the gastric digesta, whereas the ABTS assay (performed at a pH of 7.4) could evaluate the intestinal digesta. Thus, factors, such as pH, interaction of phenol compounds between pectin, protein or fat or Fe and chemical structure of the phenolics (71), method of extraction for total phenols, solvents ratio, and temperature, could affect the release of free phenols and the antioxidant capacity (67–69).

## Conclusions

This study demonstrated that fermentation of a smoothie composed of pineapple and chayote leaves with *L75* and *W64* increased the total phenol and carotenoid contents. The storage of the fermented smoothies at 4°C for 7 days could encourage continuous biochemical activities of fermenting LAB cultures. Fermentation with *L75* enhanced the level of free soluble antioxidant (total phenols) crossing a cellulose membrane (dialysis) and thus be potentially available for further uptake. However, further investigations on phenolic and carotenoid fractions with Caco-2 cellular models must be performed to confirm the uptake of phenolic and carotenoid components. Based on the finding of this study, the fermentation with *L75* can be recommended to local food manufacturers in Réunion Island to improve the functional benefits and accessibilities of total phenols in chayote leaves and pineapple smoothies. Consumer's overall acceptability for marketing is higher for the smoothie composed of pineapple and chayote leaves fermented by *L75* after 7 days of storage at 4°C than for the non-fermented sample at day 0. The metabolomics profile characterization of the fermented smoothies will be necessary to gain further knowledge.

## Data Availability Statement

The original contributions presented in the study are included in the article/supplementary material, further inquiries can be directed to the corresponding author/s.

## Author Contributions

MM performed the experiment, generated the data, and wrote the first draft of this manuscript. SA validated the data, improved the write up of physicochemical, and sensory parameters related to fermentations. FR was responsible for fermentation, conceptualized the research, and visualized and validated the data for physiochemical properties responsible for *in vitro* digestion. The research collaborator and co-supervisor provided the editorial support. CG was responsible for *in vitro* digestion analysis, methodology, and training. DS conceptualized the research, supervised MM, and improved the article further. All authors contributed to the article and approved the submitted version.

## Conflict of Interest

The authors declare that the research was conducted in the absence of any commercial or financial relationships that could be construed as a potential conflict of interest.
